# Situation, Impacts, and Future Challenges of Tobacco Control Policies for Youth: An Explorative Systematic Policy Review

**DOI:** 10.3389/fphar.2019.00981

**Published:** 2019-09-09

**Authors:** Chhabi Lal Ranabhat, Chun-Bae Kim, Myung Bae Park, Mihajlo (Michael) Jakovljevic

**Affiliations:** ^1^Policy Research Institute, Kathmandu, Nepal; ^2^Institute for Poverty Alleviation and International Development, Yonsei University, Wonju, South Korea; ^3^Manmohan Memorial Institute of Health Science, Kathmandu, Nepal; ^4^Department of Gerontology, Pai Chai University, Daejeon, South Korea; ^5^Department of Preventive Medicine, Yonsei University Wonju College of Medicine, Wonju, South Korea; ^6^Department of Global Health Economics and Policy, University of Kragujevac, Kragujevac, Serbia; ^7^Division of Health Economics, Lund University, Lund, Sweden

**Keywords:** tobacco control, smoking, policies, youth, preventive measure, tobacco products

## Abstract

**Background:** Tobacco use in youths is a major public health challenge globally, and approaches to the challenge have not been sufficiently addressed. The existing policies for tobacco control are not well specified by age.

**Objective:** Our study aims to systematically investigate existing tobacco control policies, potential impacts, and national and international challenges to control tobacco use targeting the youth.

**Data sources:** We used the statistics of the Global Youth Tobacco Survey (GYTS), studies, and approaches of tobacco control policies targeting youth. Considering country, continent, age, and significance, PubMed, Health Inter-Network Access to Research Initiative (HINARI), Scopus, the Cochrane Library, Google, and Google Scholar were searched. The related keywords were tobacco control, youth, smoking, smoking reduction policies, prevalence of tobacco use in youth, classification of tobacco control policies, incentives to prevent young people from using tobacco, WHO Framework Convention on Tobacco Control (FTCT), etc. The search strategy was by timeline, specific and popular policies, reliability, significance, and applicability.

**Results:** We found 122 studies related to this topic. There were 25 studies focusing on situation, significance, and theoretical aspects of tobacco control policies associated with youth; 41 studies on national population polices and challenges; and 7 studies for global challenges to overcome the youth tobacco epidemic. All national policies have been guided by WHO-MPOWER strategies. Increases in tobacco tax, warning signs on packaging, restriction of tobacco product advertisements, national law to discourage young people, and peer-based approaches to quit tobacco are popular policies. Smuggling of tobacco products by youth and ignorance of smokeless tobacco control approach are major challenges.

**Limitation:** Our study was flexible for the standard age of youth and we were not able to include all countries in the world and most of the studies focused on smoking control rather than all smokeless tobaccos.

**Conclusion:** The policies of tobacco control adopted by many countries are based on the WHO Framework Convention on Tobacco Control but not necessarily focused on youth. Due to the physical and economic burden of tobacco consumption by youth, this is a high priority that needs to be addressed. Youth-focused creative policies are necessary, and more priority must be given to tobacco prevention in youth. Tobacco control should be a social, public health, and quality-of-life concern rather than a business and trade issue.

**Implication of key findings**: There is limited research on how and in what ways tobacco control policies reach young people and their engagement with these policies from physical, physiological, and psychological aspects. Analysis of these aspects, popular polices practiced in different countries, and creative strategies support the need to review current practices and future ways to discourage youth from tobacco use.

## Introduction

Tobacco consumption is a major challenge for the 21st century because tobacco-related deaths are increasing, destroying the young generation and promoting an environmental threat. Globally, tobacco has killed 100 million people in the 20th century, much more than all deaths in World Wars I and II combined, and tobacco-related deaths will number around 1 billion in the 21st century if current tobacco use patterns continue (Eriksen et al., 2015). Of the 100 million projected tobacco-related deaths over the next 20 years, about half will be of people in the productive ages of 35–69 (Centers for Disease Control and Prevention, 2000). Regardless of many national bans on tobacco sales to minors, approximately 25% of people under 18 years old are using tobacco and 12.6% are using more than two types of tobacco products (Arrazola et al., 2014). There are multiple impacts (economic, health, social, family, and peer groups) of tobacco use in youth because they are losing high amount of money, as the tax on tobacco increases year by year, risk factors for many disease, vulnerability to alcohol use and drugs, and copying his/her tobacco use by juniors in schools and sibling in family put them at risk for tobacco use.

There are a variety of programs and policies for tobacco control, but policy analyses on age-specific tobacco control are very rare. It is important because the resources, efforts, and approaches to quitting tobacco for people 60 years of age and those 16 years age do not have a similar impact. A study pointed out that there is a need for a comprehensive multifaceted approach to tobacco control policies for youth (Grimsaw and Stanton, 2017; Cancer Council., 2017a). There is a need to observe that as a foundation and productive age group, youth should be a high priority because interventions would be cost-effective and more productive to family and the nation. Moreover, approaches to controlling tobacco in youth are easy in comparison with late adults and the elderly because nicotine addiction in the late stage is difficult to overcome. From the point of its effectiveness, preventive and nominal remedial approaches are sufficient for youth. In contrast, more resources and complex medical approaches are necessary in late-stage addiction. Likewise, different kinds of medical risk can be prevented in the early stage (youth) but are hardly possible in the late stage. Previous studies, research, policies, and programs are not clearly distinctly age-specific tobacco control approaches, and in our study, we explore the different dynamics of tobacco control policies focusing on youth.

### Prevalence of Tobacco Use Among Youth

Tobacco use among youth remains a major public health concern worldwide. Globally, there are about 1.2 billion smokers, of whom more than 50% are young people; the prevalence varies by region, country, and gender (Khuder et al., 2008; Lim et al., 2010; Al-Sadat et al., 2010). By gender, smoking among boys (16%) is almost three times than that among girls (6%) globally. In the West Pacific, the prevalence of smoking among boys (18%) is four times than that among girls (4%), whereas in the United States and Europe, the gap between boys and girls is less than double. Smokeless tobacco is also gaining popularity and is currently used globally by 8% (6% in boys and 2% in girls). The highest proportion of girls using smokeless tobacco (17%) is found in the West Pacific and the lowest (2%) is found in Europe (World Health Organization., 2012). India, with 327 million adolescents, has one of the youngest populations in the world; the number of adolescents using tobacco is approximately 21% of the country’s population (Vidhubala et al., 2014). Since 1980, large reductions in the estimated prevalence of daily smoking have been observed at the global level for both boys and girls (e.g., in the United States, about 10% from 1980 to 1990 of 12th-grade students), (Nelson et al., 2008) but because of population growth, the gross number of smokers has increased significantly (Ng et al., 2014). According to the Global Youth Tobacco Survey (GYTS) 2011, the top three countries for tobacco consumption rates were Papua New Guinea (43.8%), Chile (31.5%), and Lithuania (30.8%), while Cambodia consumption rate was the lowest (0.2%) (World Health Organization, 2011). Prevalence of youth tobacco use reduces life expectancy (Ranabhat et al., 2018; Ranabhat et al., 2019). Socially, youth from disadvantaged groups are more vulnerable to smoking because of their social context (Hefler and Chapman, 2014). Despite the variation in statistics between different classifications of youth, youth smoking is a major threat in every aspect.

### Understanding Youth

Youth is a critical and foundation period of human life; however, there is no consistent definition. Youth is the time of life when one is young, but often means the time between childhood and adulthood (maturity) (Walker et al., 2013). Around the world, the English terms youth, adolescent, teenager, kid, and young person are interchanged, often meaning the same thing (Konopka, 1973). The United Nations has defined the contextual definition of youth: age between 15 and 24 by UN secretaries, UNESCO, and ILO; age of 15–32 by UN habitat; age of 10–24 by UNFPA and WHO; child until 18 by UNICEF; and age between 15 and 35 by the African youth charter (United Nation., 2016). Beyond this, different countries have defined the youth in their context, and we have used the term youth as a wide concept as used by different scholars and organization. The focus of this study is on tobacco control policies applicable for youth despite the age variation.

### Youth Period and Risk for Tobacco Use/Smoking

There are theoretical and empirical studies about the risk of youth tobacco use/smoking. Youth may initiate smoking by i) social learning theory; youth are eager to try something new, i.e., attention, retention, motor reproduction, and reproduction and motivation process; (Bandura and McClelland, 1977) ii) psychological development theory; they decide whether they should use or not, i.e., age, self-control, academic achievement, growth trend, and distal structure (parents or peer); (Jessor and Jessor, 1977) iii) behavior theory; the attitude of acceptance and to continue or not, i.e., person’s behavior is a function of behavioral intention, which is determined by attitude toward the act; (Ajzen and Fishbein, 1970), and iv) self-conceiving theory; every activity of human is determined by self-conception (Rosenberg, 1986). Blum has explored different aspects of human emotions, motivation, and perceptions, particularly during the period of youth (Blum, 2009). Due to their age, physiological changes, and family and social environments, youth often perpetuate smoking practices, and they ultimately become addicted. Indeed, youth tobacco use is a primary source of substance use and other social deviations. High school male students who have smokers in family and school, smoker friends, media and advertisement influence, and easy access to the purchase of cigarettes are factors to youth smoking; smokers often lack self-control even though they know smoking is harmful (Simons-Morton et al., 1999; Alexander et al., 2001; Ertas, 2007).

### Key Questions

KQ1—What is the current situation of youth tobacco use in terms of prevalence and control policy pattern?

KQ2—What are the major and popular policies to control tobacco in the national and global context targeting youth?

KQ3—What could be the best creative policies for tobacco control for youth?

### Objective of the Study

The aim of this study is to systematically investigate the situation regarding youth tobacco use, existing policies, effectiveness, and challenges in the national and global context.

## Methods

### Eligibility Criteria

We focused precisely on tobacco control policies, targeting youth, policies adopted by countries, challenges to implementing those policies, publications in English, and availability of latest prevalence data.

Studies were included based on the following PICOS strategies.

1. Population: Youth population and age limit vary between countries.
2. Intervention: Popular and successful intervention policies to control tobacco use targeting for youth.
  - Disconnect youth from tobacco
  - Smoking/tobacco use cessation
3. Comparison: Comparison of tobacco control policies by country and intervention time duration.
4. Outcomes: Prevalence of tobacco use before intervention and after intervention.
5. Study types: Interventional studies, surveys (cross-sectional and follow up), cohort and randomized control trials, core contents related to study from official web pages and some gray materials.

Exclusion:

1. Did not meet the inclusion criteria.
2. General tobacco control not applicable for youth; data available are not in English; mixed studies with tobacco use; coffee, alcohol, and other substance use/abuse; and data with mixed/overlap of age (youth and adult/youth and elder).

**Figure 1:**
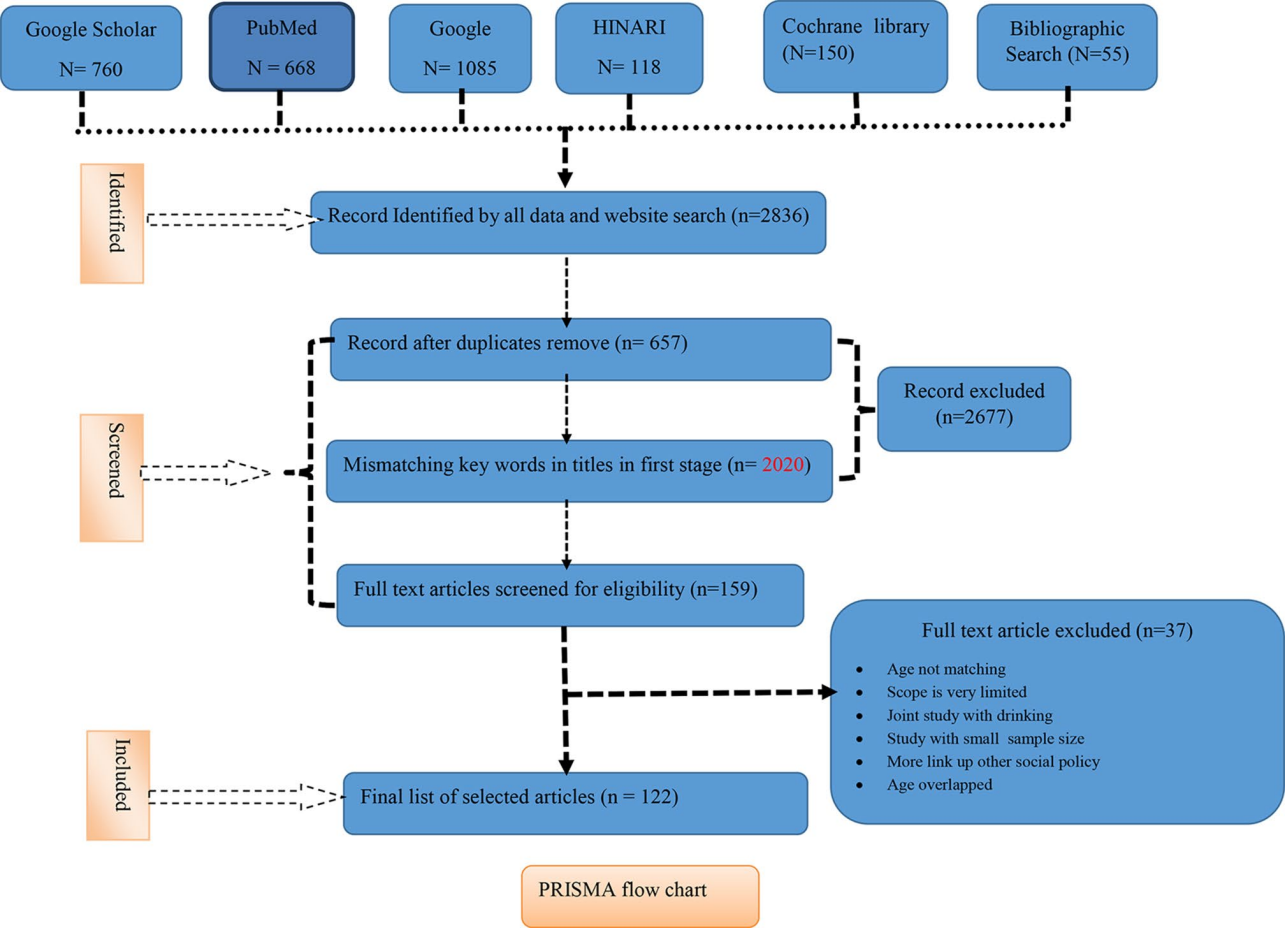
PRISMA flow chart of research strategy.

The details of excluded data with reason are available in the PRISMA flowchart (**Figure 1**).

### Data Searching Strategy

Literature search strategies were developed using Medical Subjects Headings (MeSH) terms and keywords. Different health and social science search engines and databases were used (PubMed, Scopus, the Cochrane Library, HINARI, Google, and Google Scholar) to find sources regarding global and national policies mostly focusing on youth tobacco use. We used single, double, or multiple MeSH terms, free text, and specific terms under a subheading to identify relevant studies from the online data sources. Search strategy also included content, synonyms, year, and country names (**Box 1**). We downloaded and analyzed relevant journal articles, books, survey results, analytical views related to the WHO FCTC, and unpublished reports.

Box 1 **Electronic data search strategy.**

Terminology used for search

Tobacco control policies, Smoking reduction policy, Youth and smoking, Tobacco use by adolescents , Comparative studies of tobacco use by boys and girls, Systemic review of tobacco control policies, Classification of tobacco control policies, Incentives on preventing and cessation of smoking, Legal provision for tobacco control, Effectiveness of WHO FCTC, Interventions for tobacco control on youth, MPOWER strategies, tobacco control policies by country continent, age gender, Effectiveness of MPOWER etc.

PubMed Search Options

- Search by: Terminology mentioned above
- Search Details: Single, double or multiple MeSH terms as mentioned in terminology
- Article type: All
- Text availability: Abstract and all text
- Publication date: Any time
- Complementary search: Similar articles

Scopus search options

- Search by: Subject area and title
- Display options: All available
- Source type: Journal, Book Series and Conference preceding

Cochrane library

- Search by: Key words and titles
- Date: before 2005 and between 2005 – 2019
- Language: English
- Search folder: Cochrane review, Cochrane protocol, Trial and Special collection

Google Scholar

- Search terminologies: As mentioned above by key words and titles
- Time: Any times
- Sort by: Relevance
- Included: Related citations

Health Inter Network Access to Research Initiative (HINARI)

- Search terminologies: As mentioned above by key words and titles
- Content type: Journal article, Publication, Book Chapter, Conference preceding, Data, Government documents
- Publication date: Before 2005 and between 2005 – 2019
- Discipline: Medicine, Public health, Policy
- Language: English

Google

- Key words: As mentioned above by key words and titles
- Display: all
- Purpose: Screening key titles

### Data Source Management

We, all authors, established the inclusion and exclusion criteria and data search strategies. CR and MBP searched all data and CBK and MJ verified those data. There were some data that were ambiguous and less relevant to our study but we decided to include some in our study.

### Ways of Screening and Selection of Data Sources

The title of the study was screened by search engines using major keywords and titles. Many times, search engines were used to find the appropriate titles. After that, the titles were selected. In a second step, abstract and full-length studies were selected by database search engines. In the third stage, full-length articles were screened based on inclusion criteria. Studies were assessed using criteria developed, for example, effective for public health implications, representativeness of study samples, comparability, credibility of data collection tools, and attributability to the intervention. An additional criterion of “generalizability” assessed whether findings were likely to be transferable at a global, regional, or national level. Particular attention was paid to internal and external validity; important quality and validity issues are discussed alongside study results.

### Quality Assessment

For quality assessment of resources, we used the Effective Public Health Practice Project (EPHPP) Quality Assessment Tool for Intervention (Higgins and Green, 2011) as reference. All research studies applied in this paper were searched and screened by two authors (Chhabi Ranabhat and Myung-Bae Park), with any disagreement resolved by consensus or arbitration of authors during meetings. For the risk of bias, we considered only selection bias and data collection method.

## Results

### Search Outcome

The cumulative total records found in our search screen were 2,836 from PubMed (*n* = 668) Google Scholar (*n* = 760), Google (*n* = 1,085), Health Inter-Network Access to Research Initiative (HINARI *n* = 118), Cochrane library (*n* = 150), and Bibliographic Search (*n* = 55). The total excluded 2677 records in the first stage: duplicates (*n* = 657) and mismatching keywords in titles in the first stage (*n* = 2020) were removed. From record screening, we found *n* = 159 full text sources, and out of 159 full-length articles, we excluded 37 articles due to age not matching, scope is very limited, joint study with drinking, study with a small sample size, and more links to other social policy, and we finally included 122 articles in our full study (see the PRISMA flowchart).

### Study Characteristics

**Table 1** shows the general characteristics of the study. Almost fifty percent (44.94%, *n* = 89) of our retrieved studies were about youth-targeted tobacco control in youth and most of the data (87.60%, *n* = 121) are from 2005 to 2019. We set the time frame for the study because before 2005, there was a situation of tobacco epidemic and individual countries were doing their own efforts on controlling tobacco use after 2005 (actually after signature on WHO FCTC by different countries). Regarding national policies from different continents, more than 1/3 (41.46%) are from Asian countries.

**Table 1:** General characteristics of included studies based on key questions, time interval, and region.

Characteristics	N	%
**Characteristics of studies by key questions (** ***n* = 88)**		
Situation of youth tobacco use	15	17.04
Nature of tobacco control policies focusing youth	21	23.86
Popular policies by different countries and major challenges	41	46.59
Way forward and creative policies	12	13.63
**Characteristics of studies by year (time) (** ***n* = 122)**		
Before 2005	15	12.29
2006 to 2019	107	87.70
**Characteristics of study by continent (** ***n* = 43)**		
Australia	6	13.95
Asia	17	39.53
Africa	4	9.30
Europe	4	9.30
North America	7	16.27
South America	5	11.62

### Analysis on Risk of Bias

Risk of bias analysis is not perfectly attractive with our study. We did not analyze the attrition bias because our study is a comparison of interventional studies and attrition bias occurs for randomized control trials. Moreover, we had very low influence of selection bias because all the interventions in this study were based on WHO MPOWER strategies. Studies by country and continent are not in equal proportion. We found that there were few studies in Africa, and more studies were available for Asia, but the creative policies we presented have no geographical boundaries. The number of studies for Asian countries is more than that from other continents because Asia has more than 2/3 of the world population and there are a higher number of countries compared to other continents. Most importantly, we focused on the policies related to youth and policies applicable to all age groups; gender and location were not prioritized. We applied almost all national representative survey results and global studies. There were no significant issues on data collection from the aspect on risk of bias.

### Synthesis of Result

We synthesized the results *via* a descriptive approach in three perspectives. The first one is youth-targeted current pattern of tobacco control policies and their impact. We have divided the policy pattern into two categories: i) protect the youth form tobacco use and ii) help the youth quit tobacco use. Likewise, we compared the effectiveness and challenges of tobacco control in different countries. Other important aspects are global challenges and ways on controlling tobacco use among youth.

### Patterns of Youth Tobacco Control Policies

Over the past two decades, a number of tobacco control policies have been implemented to prevent smoking initiation and encourage cessation among adolescents. To expand the fight against the tobacco epidemic, WHO introduced MPOWER, an initiative that includes six strategies: monitor tobacco use and prevention policies; protect people from tobacco smoke; offer help to people who want to quit using tobacco; warn about the dangers of tobacco; enforce bans on tobacco advertising, promotion, and sponsorship; and raise taxes on tobacco (World Health Organization, 2008). The effectiveness of MPOWER on youth is distinct. The International Agency for Research on Cancer (IARC) found that after increasing the tax and price of tobacco, there was significant reduction in tobacco use because youth are price sensitive and have a limited amount of resources (World Health Organization, 2011). Similarly, the smoke-free air law has a protective effect on young people and reduced smoking prevalence among boys of high socioeconomic status (Tauras et al., 2013). Most youth are influenced by tobacco advertisements, and according to the WHO, 24 countries have implemented a complete ban on direct and indirect tobacco advertising, promotion and sponsorship (TAPS) (World Health Organization, 2013). A study in 19 developing countries showed that there was a positive correlation between smoking and exposure to advertising (Kostova and Blecher, 2013). There were no appropriate research examining the effectiveness of offering to quit tobacco/smoking and warning of the harmful effects of tobacco applicable for youth. Therefore, in high-, (Dupont and Ward, 2002) low-, and middle-income countries, (Joseph, 2010) raising the price of tobacco and the tax on tobacco is more effective in reducing youth tobacco use than other strategies. The US Centers for Disease Control and Prevention (CDC) identified seven basic principles for tobacco control; three of these policies are relevant to youth: reducing tobacco use among adolescents; reducing the initiation of tobacco use among children, adolescents, and young adults; and increasing smoking cessation attempts among adolescent smokers (Centers for Disease Control Prevention (CDC)., 2007). A tobacco control plan in the United Kingdom has implemented three strategies to protect youth from tobacco use: reducing tobacco consumption, supporting parents and youths, and reinforcing the benefits of clean air spaces (National Health Service., 2015). Policy researchers have classified tobacco control initiatives into two groups: those intending to prevent first-time tobacco users (adolescents) and those aiming for cessation of tobacco use among current users, including both occasional and frequent users. These initiatives are based on multipronged approach, described below.

#### Disconnect Youth From Tobacco and Use Advertising Campaigns and Laws to Demotivate Youth From Tobacco Consumption

Popular policies within this guideline include cigarette tax increases, smoke-free air laws, and youth access laws (laws on sales to minors and laws against youth possession, use, and purchase)(Warner et al., 2003). Ross and Chaloupka highlighted that in the United States, higher cigarette prices reduced the probability of youth smoking, and the teen-specific perceived price of cigarettes had a negative effect on demand (Ross and Chaloupka, 2003). Similar findings have been reported by other researchers, and some have found that increased price decreased current smoking prevalence and the number of cigarettes smoked per day among youth and young adult smokers (Wasserman et al., 1991; Tauras, 2004; Levy et al., 2004). Other approaches to discourage adolescent smoking are restrictions on smoking at home, more extensive bans on smoking in public places, and enforced bans on smoking at school (Wakefield et al., 2000; Farkas et al., 2000). Sales to minors (STM) laws, which penalize merchants and retailers for selling tobacco to youth, and possession, use, and purchase laws, which punish youth themselves for possessing, using, or purchasing tobacco products, have also been applied (Rabin and Sugarman, 2001). Some studies have revealed an association between youth access and STM laws, but a sustained relationship between those laws and decreased youth smoking prevalence has been questioned. A study from Bangladesh shows that youth are more vulnerable due to a tobacco-friendly environment in school (Kabir et al., 2013). When the price increase policy was endorsed in the United States, it had a mixed impact, but a systematic review revealed no significant change in youth smoking. This law was also controversial, and cases were brought to court (Jason et al., 2005). Raising taxes on tobacco and STM laws have thus shown mixed effects, along with legal restrictions on adolescent smoking in the United States.

#### Increasing Smoking Cessation

Tobacco cessation, especially smoking cessation, depends on patterns of smoking such as age, peer pressure, influences of electronic media, and effective counseling to users. Among youth, quitting smoking has had no special program, and only 2–8% of youth smokers have attempted to quit in the Grimshaw et al., 2003 study. Hodder et al. suggested that universal school-based interventions could be an effective way to get youth to quit using tobacco and alcohol (Hodder et al., 2014).

In family and school, both approaches can be equally useful because some family members, teachers, and seniors need to quit smoking and pupils at risk must be disconnected from the first puff of smoking. Preventing youth from starting tobacco use is more effective and costs less than helping users quit, but no proper comparative studies have examined the different patterns and effects of youth tobacco control policies.

### Major National Tobacco Control Policies Intervention, Outcomes, and Challenges

**Table 2** shows the national tobacco control policies targeting youth. In 26 studies, researchers explored the overview and impact of youth-related tobacco control policies from China, India, Nepal, Thailand, Japan, South Korea, Namibia, Chad, Seychelles, Mauritius, Niger, Eritrea, Madagascar, South Africa, North Africa, France, United Kingdom, Australia, Uruguay, Panama, Colombia, Guatemala, Brazil, United States, and Canada. Likewise, in 15 studies, the challenges to control tobacco use in those countries were investigated. Seven studies were used as representative of global situations and challenges.

**Table 2:** National tobacco policies and challenges to control.

Policy intervention and outcomes by countries	Current challenges
China applied a tax increase, smoke-free policies, health warnings, media campaigns, and cessation incentives targeting youth with strong monitoring mechanisms. Youth tobacco use dropped from 10% to 3% from 2001 to 2011(World Health Organization, 2011).	The tobacco companies are not honest in implementing the warning message and pictures on packets because the letter size are small and in English (Hu et al., 2013). Only 1/4 of youth know about the harmful effect of tobacco use, and annual average cigarette sales per capita is increasing (Katanoda et al., 2013).
India and Thailand have implemented graphic health warnings on various tobacco products, and other countries in the region are in the process of implementing warnings targeting youth because it is the most cost-effective approach (World Health Organization, 2011). According to the Global Youth Tobacco Survey (GYTS), the situation of youth smoking before and after implementation was 10.8–4.4% in India and smokeless tobacco is very high.	Easy use of pocket money to purchase of tobacco and use of smokeless tobacco without tax in India (Kaur and Jain, 2011; Oswal, 2015).
Nepal government endorsed the National Tobacco Control Strategic Plan (2013–2016) special provision of youth (Ministry of Health and Population., 2013). Those provisions are raising tax on tobacco products, complete ban on advertising for tobacco products, and incentive to youth who want to quit tobacco use. The prevalence of youth tobacco use in Nepal decreased slightly from 18% to 12% in a 6-year period of time.	There is a great challenge that smokeless tobacco prevalence is increasing due to the comparative low price of cigarettes (Hawkins et al., 2018), poor implementation of health- and tobacco-related laws, (Ranabhat et al., 2019) and indigenous youth from hill areas are more likely to use tobacco (Pradhan et al., 2013).
Japan has endorsed a smoking control policy by law with four components including a smoking ban in public places, governmental offices, taxis, and schools. They have also implemented a reward strategy for youth who quit smoking, which has focused on girls and has demonstrated positive impact (Kim et al., 2013). According to WHO report 2016, youth tobacco use in 2002 was 17%, and in 2016, it reduced in 3.1% (World Health Organization, 2017).	Higher prevalence of smoking in part-time high school children, parent smoking, and alcohol use together are major challenges in Japan (Watanabe et al., 2013).
In South Korea, tobacco tax increases, mass media campaign, Health Promotion Act, and Juvenile Protection Act helped to protect youth from smoking (Do and Farooqui, 2012; Durkin et al., 2012) Youth tobacco prevalence decreased from 17.5% to 8% between 2000 and 2008 after an adjustment for gender (Korean Association on Smoking or Health., 2010).	Only 30% of cigarette packets are covered by mild and light warning signs and 30% youth suffer from secondhand smoke (Cho, 2014).
Namibia and Chad banned smoking in public places with no exceptions (i.e., no designated smoking areas), thereby creating 100% smoke-free environments and fully meeting the standards of the FCTC and its guidelines (Tumwine, 2011).	In most African countries, youth have been using cigarettes in spite of warning activities, like girls have been using in night clubs (Doku, 2010). Warning message from mass media have been highly ignored (Islami et al., 2015).
Chad further restricts smoking in vehicles carrying minors or pregnant women (Tumwine, 2011).	
Seychelles banned smoking in public places, workplaces, and public transport without designated smoking rooms. Its law does not apply to hotel rooms, although the owner may prohibit or restrict smoking (Tumwine, 2011).	
Mauritius has legislation on packaging and labeling of tobacco products, but in other countries, such legislation has only been partially adopted (Tumwine, 2011).	
Niger, Chad, Eritrea, and Madagascar have legislation against all forms of direct and indirect advertisement of tobacco (Tumwine, 2011).	
South Africa has the most comprehensive ban on tobacco advertising, promotion, and sponsorship, but the ban does not extend to advertising in books, magazines, newspapers, films, or video transmissions made outside South Africa (Tumwine, 2011) Youth tobacco decreased from 24% to 12% according to GYTS in 2011.	
The most successful approaches in North Africa include raising taxes and banning advertising (Madkour et al., 2013).	
Tobacco excise taxes and increased prices reduced tobacco consumption and prevented young people from beginning to smoke in France and some other countries. As a consequence, public health has improved (Chaloupka et al., 2011).	High prevalence of girls smoking, high socioeconomic health inequality, and parent’s smoking are becoming major challenges in the European region (Lorant et al., 2015).
Less attractive cigarette packaging, warning signs about smoking, and restrictions on advertisement have been successful in reducing youth smoking in the UK (Asamura et al., 2015).	
Stead et al. recommended nicotine replacement therapy to help youth in the UK quit smoking, citing success rates of 50–70% (Stead et al., 2008).	
First-world country using plain packaging, long anti-smoking advertisements (White et al., 2013), smoking bans in hospitals and prisons (Sullivan and Rees, 2014), and utilization of social media (Maddox et al., 2013) have all been implemented in Australia. Likewise, multi-component community education interventions and peer-based approach (Cancer Council., 2017b) are effective in influencing smoking behavior and preventing the uptake of smoking in young people. The prevalence of tobacco use by youth in 2005 was 12%, and in 2017, it declined to 6.5% (Greenhalgh EaW MH., 2019)	Australia has mostly focused on smoking cessation. Dennis Thomas has explored 21 challenges to quit smoke; youths start smoking from friends and family also but there is sufficient support to quit (Thomas et al., 2016).
Comprehensive smoke-free laws have been implemented in four countries (Uruguay, Panama, Colombia, and Guatemala), and in many cities, states, and provinces (Griffith et al., 2010). Colombia has forwarded the tobacco control law from the senate (Ruiz et al., 2009), imposed higher tobacco taxation, taken control of illegal tobacco smuggling, and reimburses medical smoking cessation interventions (Müller and Wehbe, 2008). Brazil applied tax increases, smoke-free airway laws, a mass media campaign, and a cessation program (Horta et al., 2001). The situation of youth tobacco users dropped from 11.1% to 10% between 1997 and 2011 according to the GYTS.	Tobacco smuggling is a serious problem in Latin America, and youth are at high risk and the price of cigarettes is low in Latin America and the result is youths easily start smoking using their pocket money (Muller and Wehbe, 2008).
The US applied higher tobacco taxes and well-funded tobacco prevention and cessation programs that include mass media campaigns, strong smoke-free laws, and effective regulation of tobacco products and marketing, warning pictures on tobacco packaging, a family smoking prevention law, and removed of different flavors (like chocolate) (Winickoff et al., 2011; Campaign for Tobacco-Free kids, 2013). Tobacco price and prevalence of youth smoking are inversely proportional (Carpenter and Cook, 2008) and if prices were 10% higher, 12–17 incidence (youth smoking) would be 11.9% lower (Boonn, 2016). A study showed the reduction in prevalence of youth smoking from 35% to 16% from 1999 to 2013 (Rondeau et al., 2008). Similar policies were applied by Canada and reduced the youth smoking from 25%–12% from 2002–2011 (Canadian tobacco use monitoring survey 2011; current smoking from 1985 to 2011; youth aged 15-19, 2011).	Water pipe smoking has become epidemic in North American youth; particularly in US (Soule et al., 2015). There is still insufficient strategy to overcome menthol flavor, and pictures of warning in packets and secondhand smoking, and there is increasing smoking pattern in colleges and universities.

The direction of current policies has shifted significantly after WHO FCTC in tobacco control movement. The average strength of policies adopted varies significantly by country efforts. Cultural diversity leads to different pros and cons of tobacco control initiatives to any age, gender, geography, etc., and these subtleties must be taken into account when forming policies, and the FCTC was implemented on a national and local level.

A report of the US Surgeon General in 2012 showed that the global youth tobacco use rate was decreasing satisfactorily; the rate was 28% in 2000 and had declined to 8% by 2013 (Health UDo, Services H., 2012), but that survey did not match the GYTS of 2011. WHO FCTC has a comprehensive and global impact, specific to reducing the burden of youth tobacco use, but countries are reluctant to take proactive roles and prepare specific strategies due to lack of strong monitoring by WHO (Warner and Tam, 2012). Nevertheless, taxation, clean indoor air policies, and warning labels are ranked as the highest priorities but are general approaches, and there is still a need for more specific guidelines, integrated approaches, and age-specific tobacco control initiatives.

Tobacco companies around the world are focusing on strategic plans to block the implementation of Article 11 of the WHO FCTC, which sets guidelines on packaging and labeling of tobacco products (Sebrié et al., 2010) because youth are their target group as after they have become addicted, youth become regular customers. Tobacco companies have the power and money to influence legislators (Patel et al., 2007) and challenge the government’s legislative powers through litigation (Holden and Lee, 2009). The British American Tobacco (BAT) company’s former quality controller appealed against the ban on public smoking in Uganda; however, it was not successful. The tobacco industry in Kenya went to court to challenge the Tobacco Control Act of 2007, which created challenges for public smoking ban in public places (Tumwine, 2011). Similar cases have also been seen in the US high courts without decisions, representing a challenge to implement the WHO FCTC. Tobacco companies have litigated against new cigarette labeling policies in Uruguay, Brazil, and Paraguay to stop or delay the implementation of pictorial warnings (Sebrié et al., 2010) Thus, tobacco control stakeholders and tobacco production, manufacturing, and distribution companies need to continue to fight. It shows that tobacco companies may be a negative influence on youth-friendly tobacco control policies in the future.

## Discussion

The WHO FCTC sought to substantially reduce and sustain reductions in indoor smoking and had high levels of public support and a strong public commitment with close monitoring in France, (Fong et al., 2013) China, (Levy et al., 2014) the United States and Canada, (House of Representative, USA., 2009; Levy et al., 2011; Hitchman et al., 2013) South Africa, (Reddy et al., 2013) South Korea, (Levy et al., 2010) and Brazil (Levy et al., 2012) (**Table 1**). The popular policies were tax increase, restriction on advertisement, and smoking ban in public places, but the most effective policy is raising the tax on tobacco products (Health NCfCDPaHPUOoSa., 2012) Abuse of youth to promote smoking by companies and smuggling are major challenges, particularly in Africa and South America. The above policies showed that hard policies (legal provision) could be more effective and long lasting. Some creative soft policies (program and interventions) were also equally effective but policies related to tobacco cessation are not enough. Primarily, strong monitoring and creative preventive policies led to satisfactory reduction on prevalence on youth tobacco use.

In our study, we explored the need to formulate tobacco control policies by age groups, and the vulnerable age group is the youth; there is a need for a preventive way that is cost-effective and a lesser burden to the disease both clinically and economically. There are some similar conclusions in our studies. Saleheen et al. suggested that there is need for targeted policies for youth and burden of diseases produced by tobacco (Saleheen et al., 2014). Another systematic review by Jawad with 36 studies from 15 countries yielding 125 elasticity estimates found that a 10% price increase would reduce demand by 8.3% for cigars, 6.4% for roll your owns, 5.7% for bidis, and 2.1% for smokeless tobacco (Jawad et al., 2018), mostly to youth (Levy et al., 2018). Another systematic review from 16 studies by Duncan found that a tobacco control approach in health care settings is more effective than in school and at home (Duncan et al., 2018). A policy review by Glantz concluded that e-cigarettes are replacing conventional cigarettes, but it is not a good approach to reducing nicotine dependency (Glantz and Bareham, 2018). Now, the JUUL lab’s mission is to eliminate cigarettes, but it does not relieve nicotine dependency as it only replaces traditional tobaccos; the challenge for JUUL is to eliminate nicotine dependency in the future. The tobacco control project period is also a significant factor because comprehensive tobacco control programs lead to an 8% short-term relative reduction, increasing to a 12% long-term relative reduction in smoking prevalence through the greater impact on youth smoking (Levy et al., 2018).

### Way Forward

We discussed the situation of youth tobacco use, its effectiveness, and major challenges. Now, there is the question of the way forward. UN sustainable development goal 3a indicates strengthening the implementation of the WHO Framework Convention on Tobacco Control in all countries, as appropriate. Multiple impacts of tobacco control focusing on youth directly reduce adult and premature mortality rate (Ranabhat et al., 2017). Thus, there is a need for specific policies by age because growth and development, psychology, social environment, responsibilities, and pathophysiology are not similar for all ages. In this line, there should be different policies, programs, interventions, and remedies. Similarly, the priority of tobacco control should be different because intervention for youth and the elderly does not provide similar outcomes. Other public health projects like maternal health, child reproductive health, disease control, etc., are a matter of resource allocation, and we expect measurable outcomes in each micro activity. There is no debate that tobacco prevention for youth ensures low cost and higher output. The funding organizations for tobacco control have not encouraged or disappointed because such organizations have not provided significant outcomes. If we distinguish by age, specific tobacco control, and prevention program, funding projects may revisit their policies and invest more on youth due to their economic productive life. It is possible because youth have short exposure to tobacco addiction, can make strong commitments to quit tobacco, are easy to motivate to quit tobacco, and have family and social pressure in comparison with adults and the elderly. A similar analysis can be found in *The State of Youth Tobacco Prevention and Control Spending in Alabama: Struggles, Consequences, and Solutions* report 2014 (Dunlap and McCallum, 2014).

The average strength of policies varied significantly by country. The success of tobacco control initiatives was significantly associated with the number and types of policy adopted (Wipfli and Huang, 2011). In European countries, limiting youth exposure to smoking in movies might be an effective way to prevent adolescent smoking onset (Morgenstern et al., 2013). In addition, a family smoking prevention law in the United States has reduced the prevalence of youth smoking (Ribisl, 2012). Recently, Nepal drafted a clear provision that people who use tobacco (including smokeless tobacco) will not be eligible as government employees; this policy is appealing because it directly affects unemployed youth (Sinha et al., 2012). Peer-based approach projects are best models in youth tobacco control in Australia. Youth are active users of social media such as Facebook, Twitter, YouTube, Messenger, and other apps. Creative mobilization of information technology could be useful for implementation and monitoring of youth tobacco use (Freeman, 2012). Social drama, documentary, family movies, cartoons about tobacco use impact on youth from elementary school could also discourage youth from using tobacco (Merchant, 2013). Hence, appropriate use of mass media and social network is more effective. The e-cigarette is another option for tobacco control, but regulatory issues surrounding audit of electronic cigarettes is unknown (Jovanovic and Jakovljevic, 2015). A systematic review by Park et al. concluded that the use of multimedia, tailored approaches, personalized feedback, and interactive feature programs could positively affect tobacco prevention and cessation (Park and Drake, 2015). Likewise, receptivity to tobacco advertising was significantly associated with progression toward use in adolescents (Pierce et al., 2018).

### Strengths and Limitations

This article describes tobacco control policies with a focus on the younger generation. This is an issue that is significant but often neglected. This article is intended for a wide range of readers (basic readers to policy makers, policy researchers, and other stakeholders related to tobacco control). However, this paper also has some limitations. We used the term youth as used by different authors in their articles and context than any fixed age. Here, we have used terms such as boys, girls, adolescents, elder children, and teenagers synonymously with youth. Tobacco use is focused mostly on smoking, though smokeless tobacco is also a major problem that the world also needs to face. Being a comprehensive review, all components of systematic review and meta-analysis are not applicable to use.

## Conclusion

About 1/5 of youth used tobacco globally and it has multiple impacts on health economy and family integrity. The prevalence of tobacco use is decreasing but not at a satisfactory rate. The policies of tobacco control adopted by many countries are based on the WHO Framework Convention on Tobacco Control but have not necessarily focused on youth. Due to the physical and economic burden of tobacco consumption by youth, this is a high priority that needs to be addressed. In this digital age, creative tobacco control policies focusing on youth must be applied. Successful policies of tobacco control for youth that need to be replicated by context, country, region, and gender can be recommended. Tobacco control should be a social, public health, and quality-of-life concern rather than a business and trade issue.

## Author Contributions

CR prepared the research concept and framework, collected the articles, prepared the manuscript, and overall pursued the article. C-BK verified the concept, verified the reference, and reviewed the manuscript. MBP verified all references and prepared the composition of contents. MJ reviewed the manuscript and rearranged some parts.

## Funding

This study was supported by a National Research Foundation Grant of Korea, Korean Government (NRF-2016S1A5B892520) and the Korean Medical Association (RIHP-2015-02).

## Conflict of Interest Statement

The authors declare that the research was conducted in the absence of any commercial or financial relationships that could be construed as a potential conflict of interest.

## Abbreviations

CDC, Centers for Disease Control and Prevention; FCTC, Framework Convention on Tobacco Control; GYTS, Global Youth Tobacco Survey; NRF, National Research Foundation; STM, Sales to minor; WHO, World Health Organization; IARC, International Agency for Research on Cancer; BAT, British American Tobacco.
